# Low‐Cost Custom‐Built Flow Meters for Plant Hydraulic Conductance: Validation of Accuracy, Precision, and Reproducibility

**DOI:** 10.1002/pld3.70154

**Published:** 2026-02-23

**Authors:** Jessica Samson‐Tshimbalanga, Catherine Périé, Morgane Urli

**Affiliations:** ^1^ Centre de Recherche et Innovation sur les Végétaux, Faculté des Sciences de l'Agriculture et de l'Alimentation, Pavillon de l'Envirotron Université Laval Québec Québec Canada; ^2^ Direction de la Recherche Forestière, Ministère des Ressources Naturelles et des Forêts Québec Québec Canada; ^3^ Centre d'Étude de la Forêt, Département des Sciences Biologiques Université du Québec à Montréal (UQAM) Montréal Québec Canada

## Abstract

Measurement of xylem hydraulic conductance provides access to xylem hydraulic conductivity and vulnerability to cavitation, two key traits for assessing plant sensitivity to environmental stressors. We evaluated the performance of custom‐built low‐cost pressure drop flow meters through nearly 1200 measurements across devices, laboratories, reservoir heights (10, 25 and 45 cm, used to induce pressure head and drive water flux) and PEEK tubing of hydraulic contrasting resistances. Flow meters were interchangeable, with mean differences generally < 3.5% and never exceeding 5%, with 88.9% of comparative tests showing no significant difference. Under recommended conditions (25–45 cm pressure head, downstream‐to‐upstream pressure ratio ≈0.5), precision reached 1%–7% coefficient of variation. Accuracy, assessed against reference values obtained by water displacement, was also strong, with 68% of measurements deviating by < 5% from reference values and over 78% when measured at {greater than or equal to}25 cm. At 10 cm, performance declined because sensor deviations represented a larger fraction of pressure differential, and low‐resistance PEEK tubing increased absolute but not relative error. Validated flow meters proved portable, affordable (≈2500 CAD), and reliable. Their low cost, open‐source interface, and publicly available construction protocol make them accessible to laboratories with limited resources, enabling reproducible multi‐laboratory studies of plant hydraulics and fostering international collaborations.

## 1 Introduction

Xylem hydraulic conductance reflects the capacity of the plant vascular system to transport water and solutes to growing tissues (Tyree and Zimmermann 2013). For a stem segment, hydraulic conductance ($K^{\prime }$, kg s^−1^ kPa^−1^) is defined as the ratio between flow rate (Q, kg s^−1^) and the pressure gradient (∆P, kPa) driving the flow (Cruiziat et al. 2002). Two related hydraulic traits can be derived: (i) hydraulic conductivity, that is, conductance normalized by stem segment length, and (ii) vulnerability to cavitation, that is, the loss of hydraulic conductance caused by drought‐ or freeze‐induced embolism (Melcher et al. 2012). These traits are influenced by both internal factors (xylem anatomy and sap composition) and external conditions such as drought or freezing (Cruiziat et al. 2002; Brodribb 2009; Espino and Schenk 2011). They therefore provide essential insights into species and population sensitivity to environmental stress, particularly under climate change.

Several methods have been developed to measure xylem hydraulic conductance (Sperry et al. 1988; Espino and Schenk 2011; Melcher et al. 2012). Beyond the choice of method, critical factors such as perfusing solution, sampling handling, and the avoidance of leaks or air bubbles strongly influence measurement reliability. Among these approaches, pressure drop flow meters stand out because they are inexpensive to build and repair, easily deployable in the field, and adaptable to multiple applications (Melcher et al. 2012). In this system, the driving force is a defined pressure head between upstream and downstream reservoirs, and flow rate is inferred from the pressure drop recorded by two sensors positioned on either side of a tube of known hydraulic resistance.

We initially constructed several of these devices following the protocol of Sack et al. (2011) and adapted them to Arduino control. However, measurement accuracy was limited by battery dependence, the signal conditioning circuitry of the pressure sensors, and the low resolution of the Arduino's analog‐to‐digital converter. To overcome these issues, we developed an Arduino‐based electronic control system powered by the computer's USB port, incorporating a low‐noise supply voltage regulator. High‐precision pressure sensors with a 24‐bit digital output were selected to ensure reliable pressure readings across specified pressure and temperature ranges. These sensors were calibrated and compensated over a specific temperature range for sensor offset, sensitivity, temperature effects, and nonlinearity using an application‐specific integrated circuit (Périé et al. 2025; Urli, Périé, and Lambert 2025). Data acquisition and correction (including viscosity normalization to 25°C) were managed through a custom R Shiny interface (Chang et al. 2024; R Core Team 2025).

In this study, we tested the performance of these new custom‐built pressure drop flow meters. Specifically, we assessed (i) variability between devices; (ii) consistency between laboratories; (iii) precision, that is, closeness of replicate measurements; and (iv) accuracy, that is, closeness to reference values.

## 2 Materials and Methods

### 2.1 Xylem Hydraulic Conductance Measurements Using Custom‐Built Pressure Drop Flow Meter

Flow meters were built following the protocol of Urli, Périé, and Lambert (2025), an updated version of Sack et al. (2011), describing the instrumentation, electronic control system, and R Shiny–based data acquisition used for hydraulic measurements. Each flow meter consisted of a hydraulic circuit, two pressure sensors (Honeywell, Mississauga, ON, Canada), a temperature sensor (DFRobot, Chengdu, SC, China), and an Arduino‐based electronic control system (Figure S1). The hydraulic circuit included an upstream reservoir that generated a defined pressure head, PEEK tubing (Trajan Scientific and Medical, Ringwood, VIC, Australia at DRF; IDEX Health & Science, Oak Harbor, WA, USA, at UQAM) with known hydraulic resistance, and a downstream reservoir containing the sample, all connected by manifolds and tubing (Cole‐Palmer Canada, Québec, QC, Canada). The two pressure sensors were positioned upstream and downstream of the PEEK tubing and connected to a multiport manifold supplied by the upstream reservoir. The temperature sensor was used to monitor the perfusing solution temperature in both the upstream pressure reservoir and the downstream sample reservoir. All the sensors were connected to the Arduino‐based electronic control system. Data acquisition was managed through a custom R Shiny application (Chang et al. 2024; R Core Team 2025) available on GitHub (Urli, Lambert, and Périé 2025). This application includes automatic correction of viscosity to 25°C.

The estimated cost of the flow meter (≈2500 CAD, excluding taxes) includes the main components required to assemble a functional system (pressure sensors, tubing, and electronic data acquisition components) but excludes labor costs and standard laboratory equipment (e.g., power supply and computer) that are typically already available in most facilities. Approximately two‐thirds of this cost corresponds to the electronic components (sensors, control board, and data acquisition system). This cost could be substantially reduced by assembling the electronic components in‐house following openly available designs and documentation. However, we did not retain this option because it would have required specific electronic expertise that was not available in our laboratories. Using a commercial supplier (Kynze, Saint Bruno de Montarville, QC, Canada) allowed us to ensure proper factory calibration of pressure and temperature sensors, thereby improving measurement reliability and inter‐laboratory reproducibility. Pressure sensors were calibrated daily before measurements. Calibration was performed by bypassing the PEEK tubing and applying a series of upstream pressure reservoir heights ranging from 5 to 50 cm in 5‐cm increments to establish calibration coefficients within the R Shiny interface. Calibration was considered successful when the linear regression of applied pressure (1 cm ≈ 0.001 bar) against sensor output (in psi) yielded *R* ^2^ > 0.99.

We used a PEEK tubing where a sample of unknown conductance would have been placed in the downstream reservoir. The sample was connected to the PEEK tubing of known resistance inside the flow meter, and a pressure gradient was applied. The generated pressure drop allowed for sample conductance to be calculated.

Once steady‐state flow was achieved, flow rate through the PEEK tubing and the sample was assumed to be equal, allowing for the calculation of sample hydraulic conductance ($K^{\prime }$) according to the following relation (Equation 1):

(1) $$K^{\prime }=\frac{Q}{∆P_{\text{sample}}}=\frac{∆P_{\text{tubing}}K_{\text{tubing}}^{\prime }}{∆P_{\text{sample}}}=\frac{\frac{∆P_{\text{tubing}}}{R_{\text{tubing}}}}{∆P_{\text{sample}}}$$

where Q is the flow rate through both the PEEK tubing and the sample (assumed equal at steady state), $∆P_{\text{sample}}$ is the pressure drop across the sample (corrected for backpressure), $∆P_{\text{tubing}}$ is the pressure drop across the PEEK tubing, $K\prime_{\text{tubing}}$ is the reference conductance value of the PEEK tubing, and $R_{\text{tubing}}$ is the reference hydraulic resistance of the PEEK tubing. Backpressure correction was applied by isolating the sample and the downstream pressure sensor using stopcocks to measure the backpressure after the pressure head was removed.

Data validation was carried out during measurements using procedures integrated into the R Shiny application to ensure reliability. The application verified that the measurement range, defined as the ratio of downstream to upstream pressure sensor readings, fell between 0.2 and 0.8, with an optimum at 0.5, in accordance with established protocols. Measurement stability (i.e., achieving a steady‐state flow) was assessed by ensuring the data remained stable (coefficient of variation or CV < 0.05) over the final 300 s of recording, which also constituted the minimum duration required for a valid measurement. Any data point that failed one or more of these validation criteria was excluded from further analysis.

### 2.2 PEEK Tubing Reference Values

Reference hydraulic resistance value of each individual PEEK tubing (PEEK tubing ID) was determined to (i) have the resistance reference value of the PEEK tubing used inside the flow meter and (ii) compare the measured conductance of the sample PEEK tubing to its reference conductance $\left(K\prime_{\mathrm{ref}}\right)$. Reference resistance was determined using the water displacement method. A 1 mL 1/100 graduated pipette was connected at the manifold outlet, and flow was generated using a series of upstream pressure reservoir heights (45, 35, 25, 15, and 5 cm). Flow rate was calculated from displaced volume and time. Resistance was computed as applied pressure divided by flow rate, accounting for diameter variation more accurately than the Hagen–Poiseuille equation (Sack et al. 2011). Each PEEK tubing was measured at least three times; mean resistance values were adjusted to 25°C. Reference hydraulic conductance ($K\prime_{\mathrm{ref}}$) values were subsequently calculated as the inverse of resistance.

### 2.3 Experimental Design

PEEK tubing IDs with known conductance were used as samples to assess the accuracy, precision, and repeatability of the conductance measurements obtained with custom‐built pressure drop flow meters. Three colors of PEEK tubing were used in this experiment (Table S1), each color corresponding to a nominal internal diameter (ø): yellow (ø ≈ 0.175 mm), blue (ø ≈ 0.250 mm), and orange (ø ≈ 0.500 mm). These tubing internal diameters were selected to encompass the range of hydraulic conductance values observed in conifer stem segments, allowing the system's performance to be tested across the relevant biological spectrum. The same tubing color was used both inside the flow meter and as the sample to minimize measurement error and to ensure measurements were made within the optimal range (downstream‐to‐upstream pressure ratio close to 0.5) as recommended by Melcher et al. (2012).

Five flow meters were constructed: Three were installed at the Direction de la Recherche Forestière (DRF) laboratory and two at the Université du Québec à Montréal (UQAM) laboratory. The same perfusing solution composed of degassed and filtered (0.1 μm) ultrapure water (Barnstead E‐Pure, Thermo Scientific, Waltham, MA, USA, at DRF; Milli‐Q, Avantor Inc., Radnor, Pennsylvania, USA, at UQAM) with 10 mM KCl and 1 mM CaCl_2_ was used in both laboratories.

#### 2.3.1 Flow Meter Comparison

To evaluate the measurement variability between devices under standardized conditions, three flow meters were tested using a single blue PEEK tubing ID pair (resistance sample: b1–b2) at a fixed upstream pressure reservoir height of 25 cm in the same laboratory (DRF). At least 15 measurements were performed for each device.

#### 2.3.2 Inter‐Laboratory Comparison

To assess consistency between laboratories, hydraulic conductance measurements obtained at DRF and UQAM were compared for one PEEK tubing ID combination per color from each laboratory, resulting in six pairs (resistance‐sample: y1–y4, Y4–Y2, b1–b4, B4–B2, o1–o3, and O4–O2). At least five measurements were achieved for each of the six pairs (two pairs per color) in both laboratories at upstream pressure reservoir heights of 10, 25, and 45 cm.

#### 2.3.3 Accuracy and Precision Analysis

This experimental design resulted in combinations of three tubing colors, three flow meters, and four pressure heights at DRF and three tubing colors, two flow meters, and three pressure heights at UQAM. The upstream pressure reservoir height was manually adjusted to 10, 15, 25, or 45 cm during measurements. Each laboratory tested at least four different PEEK tubing ID samples per color to account for variations in conductance between individual tubes. For each combination of flow meter, tubing color, PEEK tubing sample, and pressure height, approximately five replicate measurements were obtained, except at the 15 cm pressure height, which was only measured at DRF. We added to this dataset measurements obtained in comparison experiments that were compatible with the overall bias and precision analyses.

### 2.4 Data Analysis

#### 2.4.1 Data Validation

Raw data were filtered to exclude potential user manipulation errors by applying predetermined conductance thresholds, derived from reference values for each tubing color: < 0.1 (yellow), < 0.15 (blue) and < 1.5/1.65 kg s^−1^ kPa^−1^ (orange, DRF/UQAM). Note the slightly higher threshold for orange tubing at UQAM reflected the higher reference values observed at this laboratory (see Section 3).

#### 2.4.2 Flow Meter Comparison

Flow meter comparison was assessed by calculating mean relative bias between different flow meter devices and comparing coefficients of variation of repeated measurements. Flow meter performance was assessed by calculating the mean relative bias of each device and by comparing the coefficients of variation of repeated measurements across different flow meters. Mean relative bias by device was calculated as (Equation 2)

(2) $$\text{Relative bias}=\frac{\bar{K^{\prime }}-K_{\mathrm{ref}}^{\prime }}{K_{\mathrm{ref}}^{\prime }}\times 100$$

where ${\bar{K}}^{\prime }$ is the mean of hydraulic conductance measurements by device and $K\prime_{\mathrm{ref}}$ is the reference conductance value of b2 PEEK tubing.

Inter‐device similarity was quantified using Jaccard indices (measurement range overlap) and Bhattacharyya coefficients (distribution similarity). The Jaccard similarity index (JSI) was calculated as (Equation 3)

(3) $$\mathrm{JSI}=100*\frac{\left|A\cap B\right|}{\left|A\cup B\right|}$$

where A and B represent the measurement ranges [min, max] for each comparison, $\left|A\cap B\right|$ is the length of the intersection, and $\left|A\cup B\right|$ is the length of the union. The Bhattacharyya coefficient was calculated as (Equation 4)

(4) $$\mathrm{BC}=100*\sum_{i}\sqrt{p1_{i}*p2_{i}}$$

where $p1_{i}$ and $p2_{i}$ are the normalized probabilities of the ith bin for each distribution. Distributions were binned using 15 equally spaced intervals covering the combined range of both distributions' measurements.

#### 2.4.3 Inter‐Laboratory Reproducibility

Inter‐laboratory reproducibility was assessed by comparing the distributions of measurements obtained at DRF and UQAM using two‐sample Kolmogorov–Smirnov (KS) tests. For each combination of tubing color and upstream pressure reservoir height, the KS test was applied to evaluate whether the measurement distributions differed significantly between the two laboratories. This non‐parametric test compared the cumulative distribution functions of two samples (*n* = 15 each) and identified the maximum distance between them, providing a sensitive measure of differences in shape, location, and spread. Statistical significance was assessed at a threshold of *p* < 0.05. In addition, for each laboratory‐specific condition, mean differences and coefficients of variation were calculated to quantify bias and relative variability.

#### 2.4.4 Precision and Accuracy Analysis

Measurement precision—how close the measurements were to each other—was evaluated for each combination of tubing color and upstream pressure reservoir height. For every such combination, the spread of conductance values was assessed by calculating the range between the 5th and 95th percentiles to exclude extreme values, allowing for meaningful comparison between measurement conditions regardless of individual PEEK tubing ID. In addition, the CV and the standard error (STDERR) were computed as indicators of measurement repeatability and dispersion.

Measurement accuracy describing how close measurements are to reference values was assessed by comparing the hydraulic conductance measurements with the flow meters to reference conductance values derived from water displacement measurements. Reference values were used to define a reference interval for each tubing color per laboratory. For a tubing color, all corresponding PEEK tubing IDs were used to determine the full range (maximum–minimum) and the mean of the reference conductance values. Rather than using restrictive observed [min, max] ranges, reference intervals were defined as mean ± full range to account for volume displacement measurement uncertainty and the limited number of reference measurements per PEEK tubing ID. This conservative approach ensures robust acceptance criteria while avoiding false rejections due to overly restrictive limits.

A conductance value measured with the flow meter was considered accurate if it fell within its color‐specific reference interval. Measurements falling outside this range were classified as outliers. Inclusion rates were calculated as the percentage of measurements falling within color‐specific reference intervals, with the complement representing the outlier rate. For detailed accuracy assessment, deviation was calculated very similarly to Equation (2), but at the individual level by subtracting the specific reference hydraulic conductance of each PEEK tubing ID from each measured conductance of the corresponding tubing. Deviations were divided by the individual reference value to obtain relative deviations. Means were calculated for PEEK tubing ID. Weighed means were used to obtain relative bias by PEEK tubing color and lab.

Visual analyses were conducted to assess if flow meters, heights, or colors of PEEK tubing (i.e., range of measurements) caused systematic bias. Individual conductance deviations in kg s^−1^kPa^−1^ ($K^{\prime }-K\prime_{\mathrm{ref}}$) were plotted to visualize bias by height, color, and lab.

All statistical analyses and graphical representations were performed in R Version 4.5.0 (R Core Team 2025).

## 3 Results and Discussion

### 3.1 PEEK Tubing Reference Values

**FIGURE 1:**
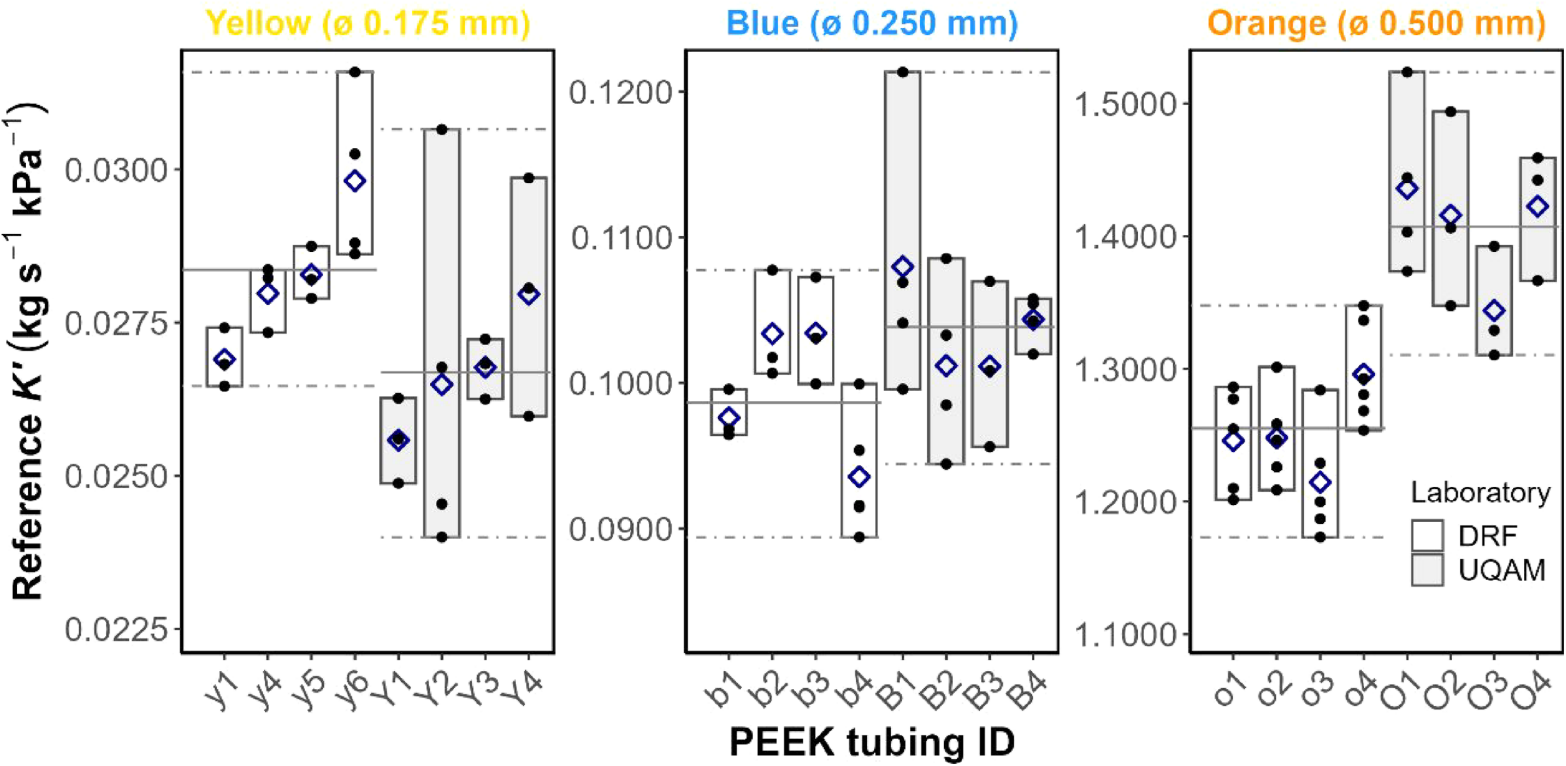
Reference hydraulic conductance values for each PEEK tubing ID by laboratory. Black dots represent individual measurements, and dark blue diamonds indicate mean values. Within each laboratory and tubing color, solid horizontal lines denote the mean, and dot‐dashed lines show minimum and maximal values (*n* = 3; except y6, Y2, B1, B2, B4, and O1 where *n* = 4; b4, o1, o2, o3 where *n* = 5; and o4 where *n* = 7). A clear hierarchical pattern emerged (yellow < blue < orange), although notable variations were observed among individual specimen within each color. These individual variations highlight the importance of characterizing each tubing specimen individually rather than relying solely on nominal diameter specifications. ALT TEXT: Three panels, ordering by tubing internal diameter of PEEK tubing (from smallest to widest, left to right). Each panel shows reference conductance against PEEK tubing ID. Reference conductance scale increases with internal diameter. Shaded rectangles indicate laboratory‐specific min–max ranges; gray shading differentiates laboratories.

As expected from their internal diameters, yellow tubing (ø ≈ 0.175 mm) exhibited the lowest conductance, blue PEEK tubing (ø ≈ 0.250 mm) was approximately four times more conductive than yellow, and orange PEEK tubing (ø ≈ 0.500 mm) was 12–14 times more conductive than blue (Figure 1). This pattern was consistent across both laboratories (DRF and UQAM).

The ranges of reference values for each PEEK tubing color showed variable inter‐laboratory agreement depending on the color examined. Blue tubing demonstrated excellent reproducibility with 83% overlap between laboratories, followed by yellow tubing with 69% overlap. In contrast, orange tubing exhibited minimal inter‐laboratory overlap of only 14%, suggesting greater systematic differences between laboratories for this specific color. Despite this limited overlap for orange tubing, the measurement ranges (max–min values) remained comparable between laboratories, indicating similar precision in measurements even when absolute values differed (Table 1). This pattern suggests that although measurement precision is consistent across laboratories, systematic differences may exist for certain tubing colors, particularly orange.

**TABLE 1:** Reference hydraulic conductance values of PEEK tubing by color and laboratory, determined with the water displacement method. Values are expressed in kg s^−1^ kPa^−1^. “Overlap” indicates the shared range between laboratories; Intersect n is the number of values falling within that range.

PEEK tubing color	DRF				UQAM				Overlap	Intersect *n*
	*n*	Mean	Min–max	CV (%)	*n*	Mean	Min–max	CV (%)		
Yellow (ø ≈ 0.175 mm)	13	0.0284	0.0265–0.0316	4.8	13	0.0267	0.0240–0.0307	7.3	0.0265–0.0307	18
Blue (ø ≈ 0.250 mm)	14	0.0986	0.0894–0.1077	5.6	15	0.1038	0.0944–0.1213	6.1	0.0944–0.1077	24
Orange (ø ≈ 0.500 mm)	22	1.2552	1.1731–1.3476	3.7	13	1.4070	1.3104–1.5235	4.5	1.3104–1.3476	5

Substantial variability was observed among individual PEEK tubing specimens within each color category. The measurement variability remained relatively consistent across colors and laboratories, with coefficients of variation ranging from 3.7% to 7.3% (Table 1 and Figure 1). Despite this overall consistency, some individual PEEK tubing specimens showed notable deviations, with O1 and O4 (orange, UQAM) having conductance values that fell outside the range observed for the same tubing color at DRF (Figure 1). Orange PEEK tubing showed the largest inter‐laboratory spread in conductance values compared to yellow and blue PEEK tubing.

These individual variations highlight the critical importance of measuring each PEEK tubing ID separately rather than relying on theoretical calculations based on the Hagen–Poiseuille equation or nominal diameter specifications. The observed differences between reference values likely stemmed from variations in tubing lengths and internal diameters during manufacturing, supporting that individual characterization of each PEEK tubing ID is essential for reliable conductance measurements, as tubing internal diameters can vary slightly from specifications (Sack et al. 2011). An advantage of using PEEK tubing is that it can be cut to better match the reference resistance of the tubing with that of the sample. In any case, the water displacement method used to assess PEEK tubing reference resistance/conductance provided simple and good flow rate measurements (Melcher et al. 2012).

### 3.2 Data Validation

A total of 37 measurements were rejected during data quality validation, representing a rejection rate of 3.09% over 1198 data points (441 from UQAM, 757 from DRF). The overall usable data rate reached approximately 97%, demonstrating the reliability of the measurement protocol when properly implemented.

Analysis of rejected measurements revealed several common sources of error: Five rejected measurements originated from UQAM likely due to downstream pressure sensor calibration error. Most rejections (32 measurements) came from DRF laboratory, with 17 measurements appearing to show hydraulic circuit leaks resulting in conductance overestimation. The remaining 15 rejected DRF measurements exhibited greatly overestimated conductance due to the downstream pressure sensor recording similar values during both active flow and backpressure measurement phases, likely caused by significant leaks, unnoticed air bubbles, or manipulation errors with stopcocks.

These findings emphasize the importance of careful system setup, proper calibration, and bubble‐free filling procedures, as any normal conductance measurement instrument requires. Careful evaluation of output data can help detect such instances and screen for problems related to calibration, bubbles, or leaks. If doubts about readings arise, swapping the sample for a PEEK tubing of known conductance can help assess results quality.

### 3.3 Flow Meter Comparison

**FIGURE 2:**
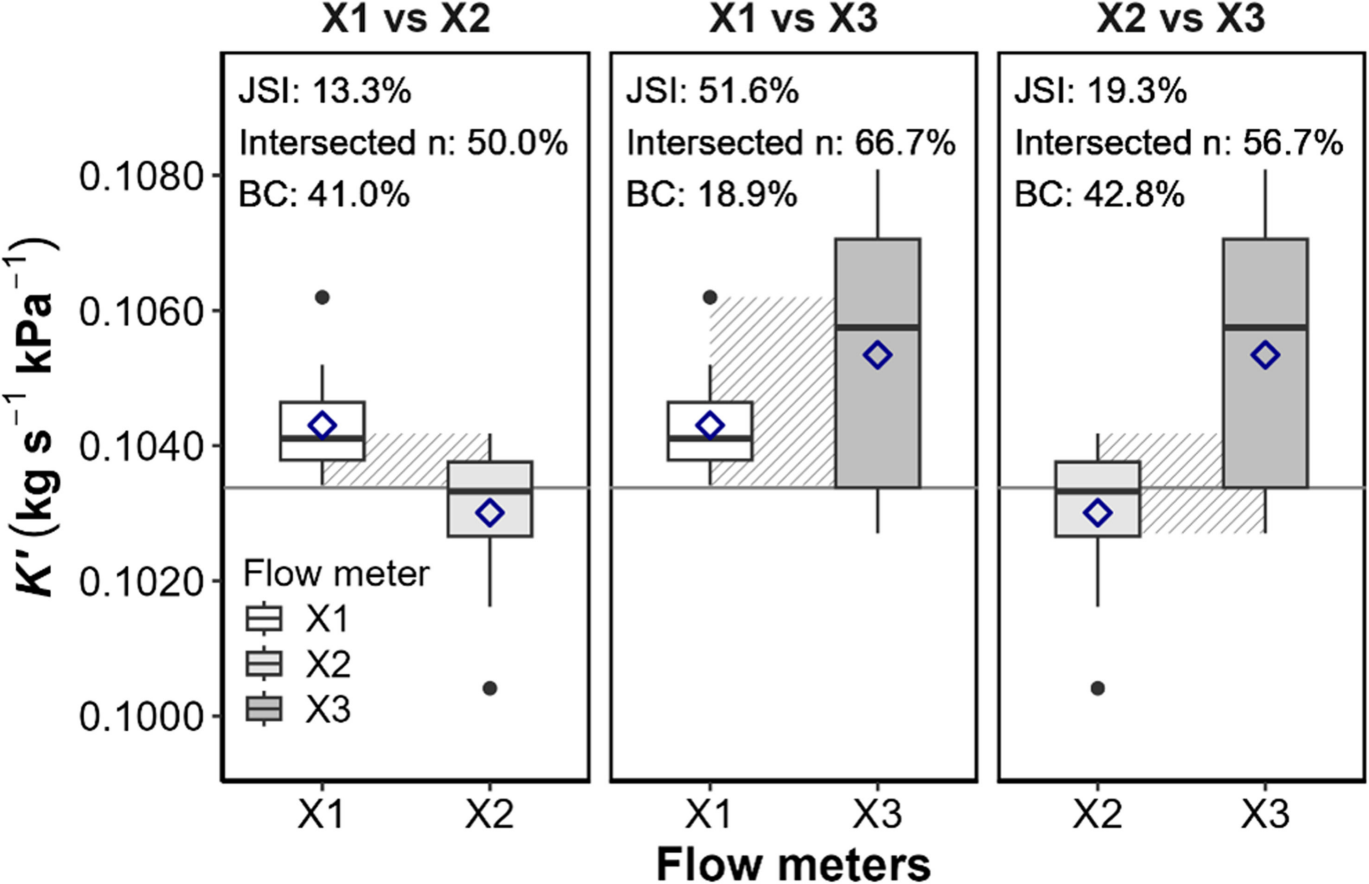
Flow meter comparison using the standardized blue PEEK tubing pair (b1–b2) at 25 cm height. Boxplots show measurement distributions (25th percentile, median, and 75th percentile; *n* = 15). Means are marked by dark blue diamonds, and outliers by black dots. Jaccard similarity indices (JSI) quantify range overlap (hatched zones), whereas Intersected *n* gives the percentage of measurements in the overlap. Bhattacharyya coefficients (BC) assess distributional similarity. Solid gray horizontal lines indicate b2 reference means determined by water displacement measurements. Results indicate that flow meters are interchangeable, with mean differences < 2.3% despite moderate distributional overlap. ALT TEXT: Three panels, each comparing two flow meters (from left to right: X1 vs. X2, X1 vs. X3, and X2 vs. X3). Boxplot are shaded in graded gray for differentiation. Numerical values of JSI, Intersected *n* and BC are shown above each panel.

Across all three flow meters, 100.0% of individual measurements remained within the tubing‐specific reference interval. The mean relative bias by device was low (from −0.36% to +1.90%), and the coefficient of variation of measurements by device ranged from 0.7% to 1.9%, indicating high internal consistency. The 5th–95th percentile range of conductance values was narrow for each device, further supporting their precision (Table S2). Jaccard indices ranged from 13.3% to 51.6%, whereas Bhattacharyya coefficients ranged from 18.9% to 42.8%, indicating moderate overlap (Figure 2). Additionally, 50.0%–66.7% of individual measurements fell within the overlapping zones between flow meter pairs, demonstrating substantial measurement compatibility. Although these values suggest some differences in distribution shape and range, differences between mean conductance values remained under 2.3% across all pairwise comparisons (1.25% for X1–X2, 0.96% for X1–X3, and 2.21% for X2–X3). Such small deviations are unlikely to affect biological interpretation or data comparability under standardized experimental conditions. These results demonstrate that flow meter identity is not a significant source of variation when tubing, pressure, and measurement protocol are held constant. Therefore, flow meters can be considered interchangeable for hydraulic conductance measurements using this experimental setup.

### 3.4 Inter‐Laboratory Reproducibility

**FIGURE 3:**
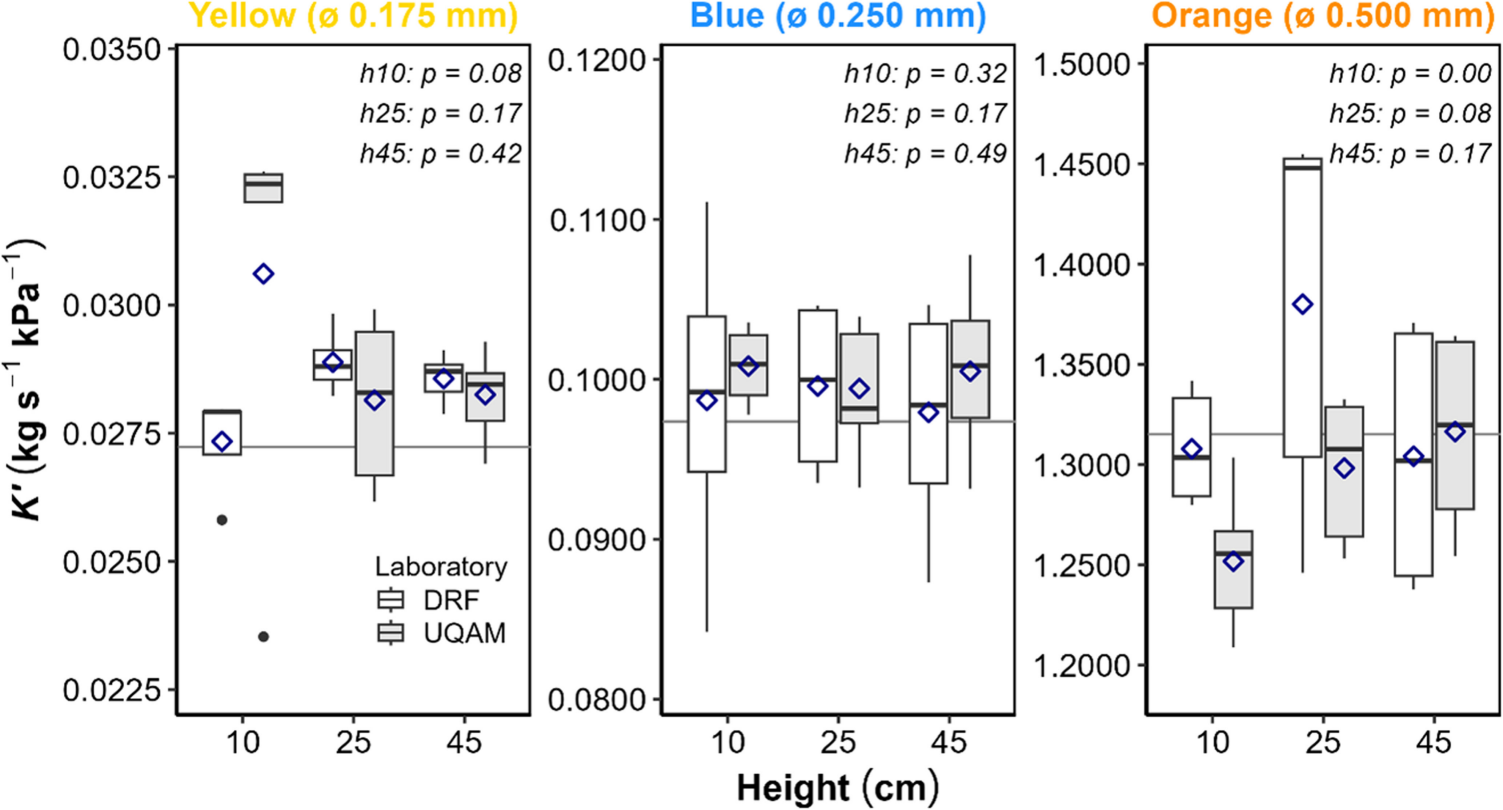
Inter‐laboratory comparison of hydraulic conductance measurements (*K*) across PEEK tubing colors and pressure heights. Boxplots show conductance measurements distribution (25th percentile, median, and 75th percentile; *n* = 10, except yellow 10 cm where both *n* = 5, blue 10 cm DRF where *n* = 7, blue 45 cm UQAM where *n* = 8, and orange 25 cm DRF where *n* = 9). Means are dark blue diamonds, and outliers are black dots. Solid horizontal lines indicate reference means by PEEK tubing color. The *p*‐values from Kolmogorov–Smirnov tests compare distributions between laboratories per height (h10 = 10 cm, h25 = 25 cm, h45 = 45 cm). Results demonstrate excellent inter‐laboratory agreement except for orange tubing at 10 cm (*p* = 0.002). ALT TEXT: Three panels, one for each internal diameter of PEEK tubing ordered from smallest to widest (left to right). Each plots conductance against pressure heights (10, 25, and 45 cm) with laboratories differentiated by gray shading. KS test *p*‐values displayed at top.

Descriptive statistics were calculated separately for each laboratory (Table S3), and visual inspection of distributions and statistical comparisons revealed strong agreement between laboratories (Figure 3). When averaging across all heights, mean conductance values differed by 1.6% for blue tubing, 3.8% for orange, and 5.0% for yellow tubing. Similarly, when averaging across all colors, mean differences by height were 5.9% at 10 cm, 3.0% at 25 cm, and 1.5% at 45 cm. Eight of nine Kolmogorov–Smirnov tests (Figure 3) indicated no statistically significant differences between distributions, with *p*‐values ranging from 0.0794 to 0.4853. A significant difference was observed for orange tubing at 10 cm (*p* = 0.0021), the only statistically significant result among all tested conditions. Yellow tubing at 10 cm showed a trend toward significance (*p* = 0.0794), indicating potential sensitivity at low‐pressure conditions. These height‐dependent inter‐laboratory differences are consistent with the precision analysis showing degraded measurement precision at 10 cm height, where CV values reached almost 9% for yellow and 3%–7% for blue and orange tubing (see Section 3.5.1 in flow meter performance assessment). The greater discrepancies observed at 10 cm can be attributed to two complementary factors: reduced measurement precision at low pressures and more noise when downstream‐to‐upstream pressure ratio is farther of 0.5 (Melcher et al. 2012). These factors explain why yellow tubing at low upstream pressure (10 cm) resulted in a high CV of 11.1% and the largest inter‐laboratory difference (11.4%), confirming the precision analysis findings of degraded performance at low pressure for low‐conductance tubing. Differences between laboratories for yellow tubing at this pressure height must be cautiously interpreted due to missing values in the dataset. For blue tubing, conductance was stable across all heights with excellent inter‐laboratory agreement (*p*‐values: 0.3206, 0.1678, and 0.4853), consistent with the acceptable precision achieved at all heights for this intermediate conductance range. Optimal performance was observed at 25 cm, showing a low coefficient of variation (CV = 4.3%) and most consistent inter‐laboratory agreement (0.2%). For yellow tubing, increasing upstream reservoir height improved stability, with most reliable results at 45 cm where CV dropped to 2.1% and inter‐laboratory difference to 1.1%. For orange tubing, increasing height from the problematic 10 cm configuration (*p* = 0.0021) to 25 cm (*p* = 0.0839) and 45 cm (*p* = 0.1678) restored excellent inter‐laboratory agreement, with best consistency at 45 cm (0.9% inter‐laboratory difference), achieving the optimal precision range of 1%–4% CV demonstrated in the performance assessment. These results demonstrate high reproducibility of hydraulic conductance measurements between laboratories based on 164 measurements, supporting protocol robustness and validating data comparability. No systematic bias depending on laboratory was detected, confirming that it is feasible to compare measurements between laboratories using these flow meters when optimal measurement conditions (≥ 25 cm height) are employed. The use of tubing and height combinations that allow the measurement range of PEEK tubing inside the flow meter to be closer to 0.5 ensures better repeatability (Melcher et al. 2012), as confirmed by inter‐laboratory comparisons and precision analysis (see Section 3.5.1 in flow meter performance assessment).

### 3.5 Flow Meter Performance Assessment

#### 3.5.1 Precision Analysis

Measurement precision showed clear patterns depending on experimental conditions (Table 2). Upstream pressure reservoir height strongly influences data dispersion, with 10 cm producing the largest ranges, standard errors, and coefficients of variation. This effect can be explained by the conductance equation (see Equation 1 in Section 2): At lower heights, upstream and downstream pressures are smaller, so a given pressure deviation represents a larger fraction of the differential, amplifying error at low‐pressure scales. Low‐resistance tubing (higher conductance) broadens variability ranges but reduces coefficients of variation, consistent with proportional error propagation. At 10 cm, small deviations have proportionally larger effects at low‐pressure heads, explaining the wider dispersion observed under these conditions.

**TABLE 2:** Precision metrics for hydraulic conductance measurements by PEEK tubing color, upstream pressure reservoir height, and laboratory. Values are based on 5th–95th percentiles to exclude extremes.

PEEK tubing color	Height (cm)	DRF 5th–95th percentiles				UQAM 5th–95th percentiles			
		*n*	Range(kg s^−1^ kPa^−1^)	STDERR (kg s^−1^ kPa^−1^)	CV (%)	*n*	Range (kg s^−1^ kPa^−1^)	STDERR (kg s^−1^ kPa^−1^)	CV (%)
Yellow (ø ≈ 0.175 mm)	10	61	0.0090	0.0003	8.6	39	0.0116	0.0004	8.9
	15	13	0.0020	0.0002	2.1	n.d.	n.d.	n.d.	n.d.
	25	62	0.0060	0.0002	4.3	39	0.0065	0.0003	6.7
	45	62	0.0038	0.0001	3.7	39	0.0039	0.0002	4.3
Blue (ø ≈ 0.250 mm)	10	54	0.0224	0.0009	6.7	38	0.0183	0.0007	4.2
	15	13	0.0222	0.0014	5.0	n.d.	n.d.	n.d.	n.d.
	25	96	0.0134	0.0004	3.7	39	0.0169	0.0008	4.8
	45	57	0.0129	0.0004	3.4	37	0.0166	0.0008	4.5
Orange (ø ≈ 0.500 mm)	10	57	0.2424	0.0058	3.4	36	0.3137	0.0118	5.2
	15	18	0.1144	0.0087	2.9	n.d.	n.d.	n.d.	n.d.
	25	56	0.0765	0.0024	1.5	39	0.1476	0.0060	2.7
	45	57	0.0833	0.0023	1.4	39	0.1539	0.0073	3.3

Tubing resistance also affected dispersion. Low‐resistance tubing (orange, higher conductance) broadened absolute variability but reduced coefficients of variation, consistent with proportional error propagation. Thus, in absolute terms, dispersion increased with conductance (orange > blue > yellow), but in relative terms, precision improved as CVs decreased with increasing conductance.

Optimal precision was obtained at 25–45 cm reservoir height. At 45 cm, CVs were 4%–5% for yellow and blue tubing and 2%–3% for orange. At 25 cm, values remained good to excellent (4%–7% for yellow, 4%–5% for blue, and 2%–3% for orange). At 10 cm, however, CVs reached 9% for yellow and 4%–7% for blue and orange, reflecting reduced measurement reliability. These results are consistent with the specified sensor accuracy (0.25%, ~0.001 bar), which makes low‐pressure conditions inherently more error‐prone.

Practical implications emerged in inter‐laboratory comparisons. The only statistically significant difference between laboratories occurred under the 10 cm condition (orange tubing, *p* = 0.0021; see Section 3.4). Unless low‐pressure heads are required to avoid embolism displacement or for other specific reasons, measurements should therefore be conducted at ≥ 25 cm to ensure robust precision.

Importantly, the flow meters described here correspond to the same custom‐built devices developed and routinely used at the DRF laboratory. Earlier version of these devices has already been applied to excised conifer stems for stem hydraulic conductivity measurements in our laboratories (Urli et al. 2023a, 2023b). This prior application on plant material provides the biological context complementary to the tubing‐based instrumental validation presented in the present study. Regarding the operating pressure range, the meta‐analysis of Urli et al. (2023a) showed that the flow meters using reservoir height to induce pressure head were extensively used on excised conifer stems under pressure gradients comparable to those recommended here (≈2–3 kPa; ~20–30 cm H_2_O). Across these measurements, no evidence of embolism displacement or refilling was observed during conductivity determination. Moreover, the same meta‐analysis of Urli et al. (2023a) comparing vulnerability curve parameters across methods revealed no systematic bias toward less negative P50 values for studies relying on hydraulic flow meter measurements. Such a bias would be expected if the applied driving forces artificially restored conductivity. Together, these results support the conclusion that the pressure range recommended here is compatible with conifer stem hydraulic measurements and does not promote artificial embolism removal.

**FIGURE 4:**
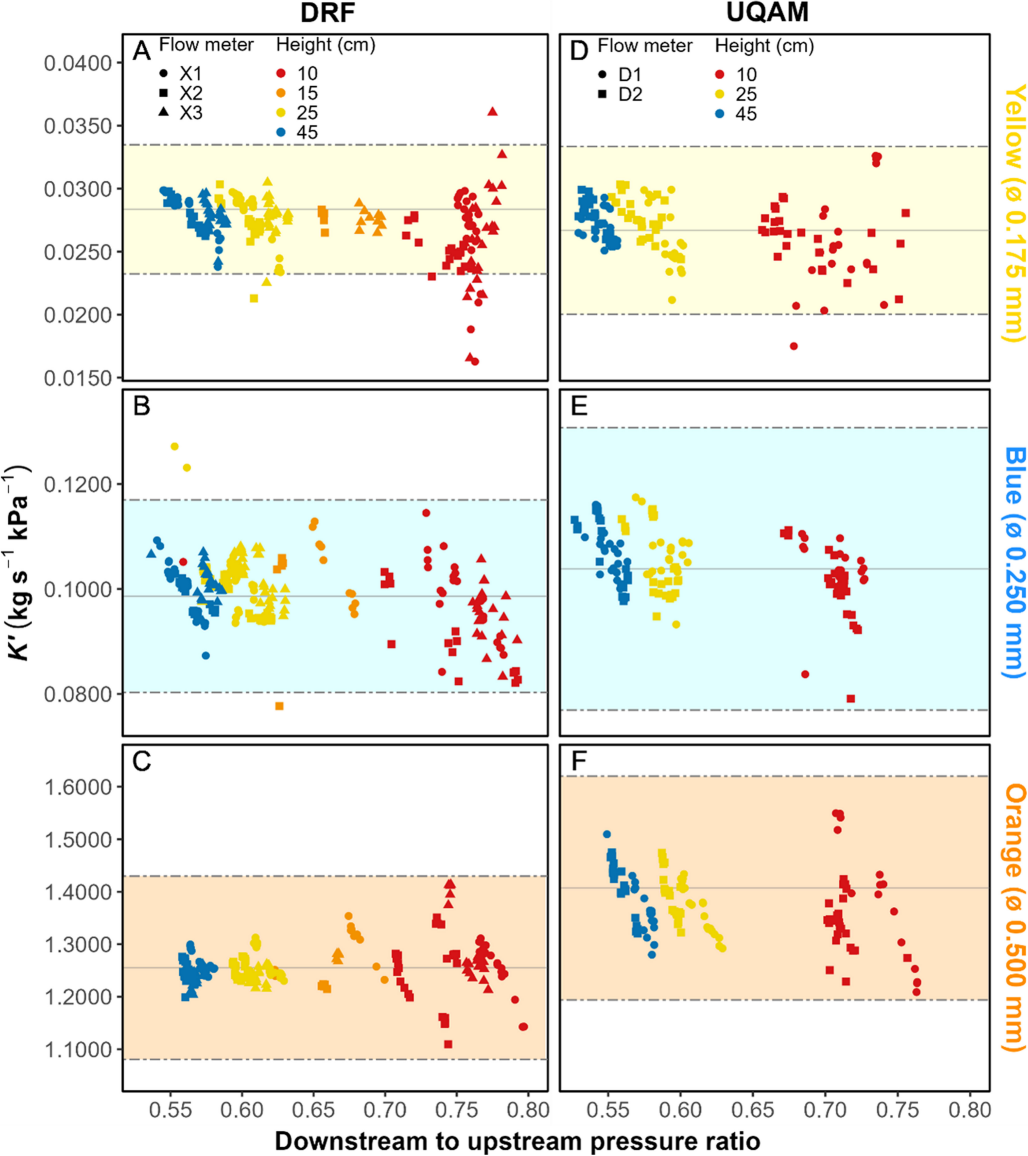
Flow meter measurement precision and accuracy assessment by PEEK tubing color, upstream pressure reservoir height, and flow meter. Points represent individual measurements colored by upstream pressure reservoir height and shaped by flow meter (DRF: yellow *n* = 224, blue *n* = 248, and orange *n* = 216; UQAM: yellow *n* = 135, blue *n* = 132, and orange *n* = 130). Solid horizontal lines represent mean reference values; dot‐dashed lines show reference intervals (mean ± range per color and laboratory). The measurement range (downstream‐to‐upstream pressure ratio) approaches the optimal 0.5 value at higher pressures. Uneven reference intervals reflect different reference measurement variability between laboratories. ALT TEXT: Combined graphs are lettered A–F. Graphs are aligned in three rows, one for each internal diameter of PEEK tubing ordered from smallest to widest (top to bottom), and two columns, one for each laboratory (DRF and UQAM). Graphs plot conductance against downstream‐to‐upstream pressure ratio. Conductance scale increases with internal diameter. Flow meters measurements are differentiated by shape of points, whereas pressure heights are color coded.

These findings also align with the theoretical prediction of Melcher et al. (2012), who showed that measurement is optimal when tubing resistance matches that of the sample, yielding a downstream‐to‐upstream pressure ratio near 0.5. Indeed, the highest water columns approached this ratio and produced the tightest grouping of measurements across tubing types in both laboratories (Figure 4).

#### 3.5.2 Accuracy Assessment

Accuracy was evaluated by comparing measured conductance values to tubing‐specific reference values. Overall, inclusion rates within reference ranges exceeded 97% for most conditions, except at 10 cm where more outliers occurred (e.g., yellow DRF 84% and UQAM 98%; Table S4). At 25 and 45 cm, inclusion rates consistently exceeded 97% across all tubing colors and laboratories, confirming robust accuracy under recommended conditions.

**FIGURE 5:**
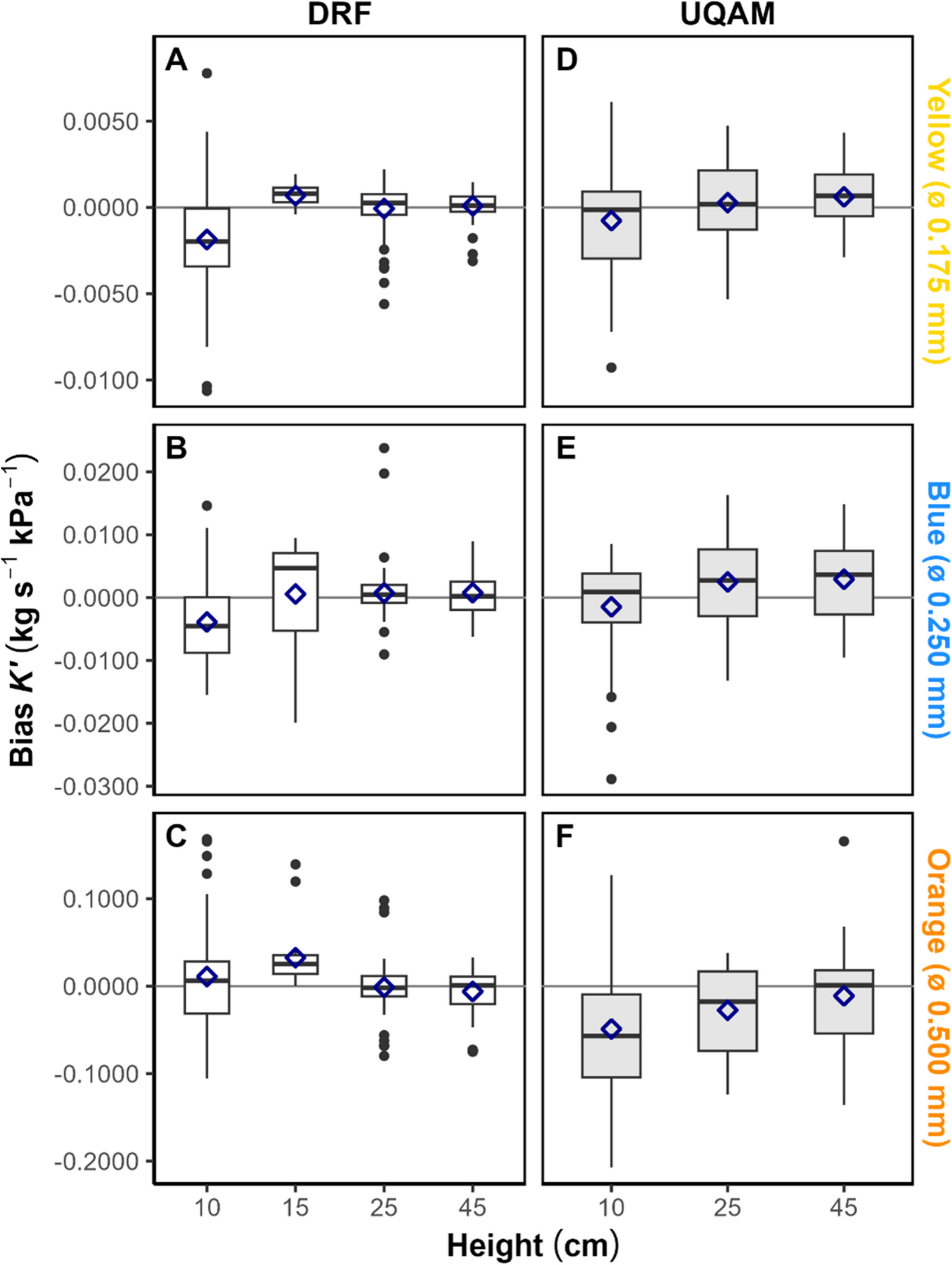
Bias in hydraulic conductance by upstream pressure reservoir height, PEEK tubing color, and laboratory. Boxplots show bias by upstream pressure reservoir height for yellow (A,D), blue (B,E), and orange (C,F) PEEK tubing for each laboratory (A–C for DRF; D–F for UQAM). Boxes show bias distributions for each color and height combination per lab with 25th percentile, median and 75th percentile (*n* = 60–70 for DRF except at 15 cm where *n* = 15–22, and blue 25 cm where *n* = 108; *n* = 40–45 for UQAM; see Table S4 for precise *n*). Means are dark blue diamonds, and outliers are black dots. Note the negative bias tendency at 10 cm height and values closer to zero at optimal heights (25–45 cm). ALT TEXT: Six panels (yellow, blue, orange × 2 laboratories). Boxplots of conductance bias against reservoir height. Gray shading differentiates labs.

Absolute mean deviations ranged from 0.13% to 8.27%, with 68% of measurements deviating by less than 5% from reference values. Accuracy improved at higher reservoir heights: 77% of measurements at 25 cm and 79% at 45 cm were within 5% of reference values. No systematic bias was detected, as deviations were both positive and negative depending on tubing ID and laboratory (Figure 5 and Table S5).

This pattern reflects the influence of pressure head on error propagation. At 10 cm, the small upstream pressure resulted in proportionally larger effects of small deviations, generating a slight negative bias (−0.050 to −0.001 kg s^−1^ kPa^−1^, with a single positive deviation of ~0.010  kg s^−1^ kPa^−1^). At 25 and 45 cm, biases were close to zero (±0.003 kg s^−1^ kPa^−1^, maximum −0.027  kg s^−1^ kPa^−1^), demonstrating the stabilizing effect of higher‐pressure heads.

Accuracy by tubing color and ID showed no evidence of systematic error. Weighted mean relative bias remained below 3% in all cases, with values ranging from −2.1% to +1.2% depending on tubing color and laboratory. This confirms that although some tubing IDs displayed larger deviations, overall flow meter measurements were unbiased and closely aligned with reference conductance values.

Together, these results indicate that measurement accuracy is strongly dependent on pressure head. Low heights (10 cm) introduce proportionally larger deviations, whereas recommended conditions (≥ 25 cm) yield highly accurate measurements across tubing colors and laboratories.

## 4 Conclusion

Hydraulic flow meters can provide information on xylem hydraulic conductance and be used to create vulnerability curves, both of which help estimate how well species will adapt to present and future environmental conditions. The custom‐built flow meters evaluated in this study provide precise, repeatable, and accurate data using a user‐friendly interface. They are low‐pressure flow meters suitable for conductance measurements, achieving optimal precision when water column height provides higher pressure (25–45 cm ≈ 0.025–0.045 bar), while still delivering steady measurements at lower settings more appropriate to avoid embolism displacement.

Key performance metrics demonstrate the reliability of these instruments: overall precision of 1%–7% coefficient of variation under optimal conditions; accuracy with 68% of measurements deviating less than 5% from reference values, increasing to over 78% at recommended pressure head heights; and inter‐device consistency with differences under 2.3% between flow meters. Most importantly, inter‐laboratory reproducibility was high, with mean differences generally below 3.5% and never exceeding 5% across all measurement conditions, and with no statistically significant differences in 88.9% of comparative tests.

These findings also highlight the importance of experimental settings. Lower‐pressure heads (10 cm) amplified small deviations, leading to greater dispersion and slight negative bias, whereas higher heads (≥ 25 cm) stabilized measurements and minimized error propagation. Similarly, absolute measurement errors increased with conductance, but relative precision improved because these errors represented a smaller fraction of the measured value.

Beyond their technical performance, these validated flow meters open new opportunities for comparative multi‐laboratory studies in plant physiology. The demonstrated reproducibility facilitates international collaborative research on tree hydraulic properties such as xylem hydraulic conductivity or vulnerability to cavitation, which are critical for predicting how plant species will respond to climate change. Their low‐cost construction (roughly less than 2500 CAD at current prices), open‐source R Shiny interface, and the availability of a complete construction protocol on the Prometheus Wiki further enhance accessibility, enabling laboratories with limited resources to conduct precise hydraulic measurements and thereby broadening the research community's capacity to address global ecological challenges.

Although PEEK tubing provided ideal controlled conditions, actual plant specimens present additional technical challenges such as variable conduit architecture, spontaneous air bubble formation, and tissue degradation that may require protocol adaptations. Additionally, validation at higher pressures and across broader conductance ranges would further extend the applicability of these flow meters.

## Author Contributions

**M.U.:** conceptualization, data curation, funding acquisition, investigation, methodology, project administration, resources, software, supervision, validation, writing – review and editing. **C.P.:** conceptualization, data curation, formal analysis, funding acquisition, investigation, methodology, project administration, resources, supervision, validation, writing – review and editing. **J.S.‐T.:** data curation, formal analysis, validation, visualization, writing – original draft.

## Funding

This work was funded by the 2030 Plan for a Green Economy (project number 112959358 led by Catherine Périé) and by a Natural Sciences and Engineering Research Council of Canada (NSERC) (RGPIN‐2024‐05901) Discovery Grant led by Morgane Urli.

## Conflicts of Interest

The authors declare no conflicts of interest.

## Peer Review

The peer review history for this article is available in the Supporting Information for this article.

## Supporting information


**Data S1:** Peer review.

**Figure S1:** Experimental setup of the custom‐built flow meter. This photograph illustrates the configuration used in the present study; detailed schematics and step‐by‐step descriptions of the flow meter are provided in the published protocol (Urli, Lambert, and Périé 2025).

**Table S1:** PEEK tubing IDs and characteristics of PEEK tubing used.

**Table S2:** Flow meter comparison: Performance metrics using standardized blue PEEK tubing combination (b1–b2 pair) at 25 cm upstream pressure reservoir height.

**Table S3:** Inter‐laboratory comparison: Descriptive statistics of hydraulic conductance measurements by PEEK tubing color, upstream pressure reservoir height, and laboratory.

**Table S4:** Measurement accuracy assessment: Inclusion rates within acceptance limits by PEEK tubing color, upstream pressure reservoir height, and laboratory.

**Table S5:** Individual PEEK tubing accuracy assessment: Relative deviations by PEEK tubing ID and relative bias by color.

## Data Availability

Data supporting this study are being deposited in the Federated Research Data Repository (Périé et al. 2025).

## References

[pld370154-bib-0001] Brodribb, T. J. 2009. “Xylem Hydraulic Physiology: The Functional Backbone of Terrestrial Plant Productivity.” Plant Science 177: 245–251. 10.1016/j.plantsci.2009.06.001.

[pld370154-bib-0002] Chang, W. , J. Cheng , J. J. Allaire , et al. 2024. “Shiny: Web Application Framework for R. R Package Version 1.9.1.” https://CRAN.R‐project.org/package=shiny.

[pld370154-bib-0003] Cruiziat, P. , H. Cochard , and T. Ameglio . 2002. “Hydraulic Architecture of Trees: Main Concepts and Results.” Annals of Forest Science 59: 723–752. 10.1051/forest:2002060.

[pld370154-bib-0004] Espino, S. , and H. J. Schenk . 2011. “Mind the Bubbles: Achieving Stable Measurements of Maximum Hydraulic Conductivity Through Woody Plant Samples.” Journal of Experimental Botany 62: 1119–1132. 10.1093/jxb/erq338. 21147811 PMC3022400

[pld370154-bib-0005] Melcher, P. J. , N. M. Holbrook , M. J. Burns , et al. 2012. “Measurements of Stem Xylem Hydraulic Conductivity in the Laboratory and Field.” Methods in Ecology and Evolution 3: 685–694. 10.1111/j.2041-210X.2012.00204.x.

[pld370154-bib-0006] Périé, C. , J. Samson‐Tshimbalanga , and M. Urli . 2025. “Low‐Cost Custom‐Built Flow Meters for Plant Hydraulic Conductance: Validation of Accuracy, Precision and Reproducibility (Data).” Federated Research Data Repository. 10.20383/103.01429. PMC12928992 41743594

[pld370154-bib-0007] R Core Team . 2025. “R: A Language and Environment for Statistical Computing.” Vienna, Austria: R Foundation for Statistical Computing. https://www.R‐project.org/.

[pld370154-bib-0008] Sack, L , M. Bartlett , C. Creese , G. Guyot , and C. Scoffoni . 2011. “PrometheusWiki Contributors.” Constructing and Operating a Hydraulic Flow Meter [WWW Document]. Accessed September 30, 2018. https://prometheuswiki.rsb.anu.edu.au/tiki‐index.php?page=Constructing+and+operating+a+hydraulics+flow+meter.

[pld370154-bib-0009] Sperry, J. S. , J. R. Donnelly , and M. T. Tyree . 1988. “A Method for Measuring Hydraulic Conductivity and Embolism in Xylem.” Plant, Cell & Environment 11: 35–40. 10.1111/j.1365-3040.1988.tb01774.x.

[pld370154-bib-0010] Tyree, M. T. , and M. H. Zimmermann . 2013. Xylem Structure and the Ascent of Sap. 2nd ed. Springer Science & Business Media.

[pld370154-bib-0011] Urli, M. , M.‐C. Lambert , and C. Périé . 2025. “UrliLab/Flowmeter‐Project: v1.3. Version 1.3.” 10.5281/zenodo.16969363.

[pld370154-bib-0014] Urli, M. , C. Périé , and M.‐C. Lambert . 2025. “An R Shiny Application for Use With an Electronic Box Flowmeter ‐ Protocol.” https://prometheusprotocols.net/function/water‐relations/hydraulic‐conductance‐and‐conductivity/an‐r‐shiny‐application‐for‐use‐with‐an‐electronic‐box‐flowmeter/.

[pld370154-bib-0012] Urli, M. , C. Périé , N. Thiffault , et al. 2023a. “On the Need to Report the Variability and Data Used in the Determination of Xylem Vulnerability Curve Parameters.” Journal of Plant Hydraulics 9: 001. 10.20870/jph.2023.001.

[pld370154-bib-0013] Urli, M. , C. Périé , N. Thiffault , et al. 2023b. “Experimental Drier Climates Affect Hydraulics and Induce High Mortality of Seedlings of Three Northern Conifer Species.” Forest Ecology and Management 544: 121127. 10.1016/j.foreco.2023.121127.

