# Supplementary material for: Low‐Cost Custom‐Built Flow Meters for Plant Hydraulic Conductance: Validation of Accuracy, Precision, and Reproducibility
Source: Plant Direct. 2026 Feb 23;10(2):e70154. doi: 10.1002/pld3.70154 (PMC12928992; doi:10.1002/pld3.70154)
Supplement: Supplementary file 2 — Figure S1: Experimental setup of the custom‐built flow meter. This photograph illustrates the configuration used in the present study; detailed schematics and step‐by‐step descriptions of the flow meter are provided in the published protocol (Urli, Lambert, and Périé 2025). [file PLD3-10-e70154-s002.docx]

**
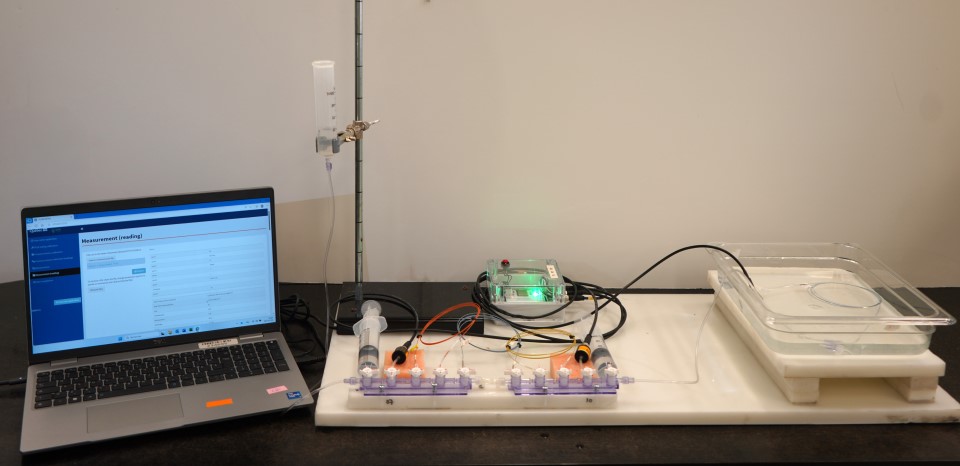
**

**Figure S1**. **Experimental setup of the custom-built flow meter. This photograph illustrates the configuration used in the present study; detailed schematics and step-by-step descriptions of the flow meter are provided in the published protocol (Urli et al., 2025a).**
