# Supplementary material for: Low‐Cost Custom‐Built Flow Meters for Plant Hydraulic Conductance: Validation of Accuracy, Precision, and Reproducibility
Source: Plant Direct. 2026 Feb 23;10(2):e70154. doi: 10.1002/pld3.70154 (PMC12928992; doi:10.1002/pld3.70154)
Supplement: Supplementary file 3 — Table S1: PEEK tubing IDs and characteristics of PEEK tubing used. [file PLD3-10-e70154-s007.docx]

**Table S1. PEEK tubing identifiers (IDs) and their characteristics.**

| Laboratory | PEEK tubing color | PEEK tubing IDs | Internal diameter (mm) | Length (cm) |
| --- | --- | --- | --- | --- |
| DRF | Yellow | y1, y4, y5, y6 | 0.175 | 152.2 to 164 |
|  | Blue | b1, b2, b3, b4 | 0.250 | 154.2 to 160.5 |
|  | Orange | o1, o2, o3, o4 | 0.500 | 154.1 to 160.5 |
| UQAM | Yellow | Y1, Y2, Y3, Y4 | 0.175 | 155.1 to 155.5 |
|  | Blue | B1, B2, B3, B4 | 0.250 | 155.1 |
|  | Orange | O1, O2, O3, O4 | 0.500 | 155.1 to 155.3 |
