# Supplementary material for: Low‐Cost Custom‐Built Flow Meters for Plant Hydraulic Conductance: Validation of Accuracy, Precision, and Reproducibility
Source: Plant Direct. 2026 Feb 23;10(2):e70154. doi: 10.1002/pld3.70154 (PMC12928992; doi:10.1002/pld3.70154)
Supplement: Supplementary file 4 — Table S2: Flow meter comparison: Performance metrics using standardized blue PEEK tubing combination (b1–b2 pair) at 25 cm upstream pressure reservoir height. [file PLD3-10-e70154-s005.docx]

**Table S2. Flow meter comparison metrics using standardized blue PEEK tubing pair (b1-b2) at 25 cm upstream pressure reservoir height. Inclusion rate is the percentage of measurements falling within the PEEK tubing reference range.**

| Flow meter | n | Mean | Relative bias (%) | CV (%) | Inclusion rate (%) | 5^th^-95^th^ percentile range |
| --- | --- | --- | --- | --- | --- | --- |
| X1 | 15 | 0.1043 | 0.89 | 0.7 | 100.0 | 0.0020 |
| X2 | 15 | 0.1030 | -0.36 | 1.0 | 100.0 | 0.0029 |
| X3 | 15 | 0.1053 | 1.90 | 1.9 | 100.0 | 0.0049 |

Reference conductance of b2 PEEK tubing (${K'}_{ref}$): 0.103379 kg s⁻¹ kPa⁻¹

Reference interval of b2 PEEK tubing: 0.096292- 0.110465 kg s⁻¹ kPa⁻¹
