# Supplementary material for: Low‐Cost Custom‐Built Flow Meters for Plant Hydraulic Conductance: Validation of Accuracy, Precision, and Reproducibility
Source: Plant Direct. 2026 Feb 23;10(2):e70154. doi: 10.1002/pld3.70154 (PMC12928992; doi:10.1002/pld3.70154)
Supplement: Supplementary file 5 — Table S3: Inter‐laboratory comparison: Descriptive statistics of hydraulic conductance measurements by PEEK tubing color, upstream pressure reservoir height, and laboratory. [file PLD3-10-e70154-s003.docx]

**Table S3. Inter-laboratory comparison of hydraulic conductance by PEEK tubing color, upstream pressure reservoir height and laboratory. Descriptive statistics are provided, expressed in kg s⁻¹ kPa⁻¹.**

| PEEK tubing color | Height (cm) | $\boldsymbol{K'}\boldsymbol{-DRF}$ | | | | | |  | $\boldsymbol{K'}\boldsymbol{-UQAM}$ | | | | | |
| --- | --- | --- | --- | --- | --- | --- | --- | --- | --- | --- | --- | --- | --- | --- |
|  |  | **n** | **Mean** | **CV (%)** | **Min** | **Max** | **Range** |  | **n** | **Mean** | **CV (%)** | **Min** | **Max** | **Range** |
| Yellow | **10** | 5 | 0.0273 | 3.4 | 0.0258 | 0.0280 | 0.0022 |  | 5 | 0.0306 | 13.0 | 0.0235 | 0.0326 | 0.0091 |
|  | **25** | 10 | 0.0289 | 1.9 | 0.0282 | 0.0298 | 0.0016 |  | 10 | 0.0281 | 5.4 | 0.0262 | 0.0299 | 0.0037 |
|  | **45** | 10 | 0.0286 | 1.4 | 0.0279 | 0.0291 | 0.0012 |  | 10 | 0.0283 | 2.6 | 0.0269 | 0.0293 | 0.0024 |
| Blue | **10** | 7 | 0.0987 | 9.4 | 0.0842 | 0.1111 | 0.0269 |  | 10 | 0.1008 | 2.2 | 0.0978 | 0.1036 | 0.0058 |
|  | **25** | 10 | 0.0996 | 5.1 | 0.0935 | 0.1046 | 0.0111 |  | 10 | 0.0994 | 3.6 | 0.0932 | 0.1039 | 0.0107 |
|  | **45** | 10 | 0.0979 | 6.5 | 0.0873 | 0.1046 | 0.0173 |  | 8 | 0.1005 | 4.7 | 0.0932 | 0.1078 | 0.0146 |
| Orange | **10** | 10 | 1.3078 | 2.0 | 1.2799 | 1.3418 | 0.0619 |  | 10 | 1.2518 | 2.3 | 1.2089 | 1.3035 | 0.0946 |
|  | **25** | 9 | 1.3800 | 6.3 | 1.2460 | 1.4548 | 0.2088 |  | 10 | 1.2982 | 2.6 | 1.2532 | 1.3324 | 0.0793 |
|  | **45** | 10 | 1.3041 | 5.0 | 1.2377 | 1.3707 | 0.1330 |  | 10 | 1.3164 | 3.6 | 1.2545 | 1.3641 | 0.1097 |
