# Supplementary material for: Low‐Cost Custom‐Built Flow Meters for Plant Hydraulic Conductance: Validation of Accuracy, Precision, and Reproducibility
Source: Plant Direct. 2026 Feb 23;10(2):e70154. doi: 10.1002/pld3.70154 (PMC12928992; doi:10.1002/pld3.70154)
Supplement: Supplementary file 6 — Table S4: Measurement accuracy assessment: Inclusion rates within acceptance limits by PEEK tubing color, upstream pressure reservoir height, and laboratory. [file PLD3-10-e70154-s004.docx]

**Table S4. Accuracy assessment: inclusion rates of measurements within acceptance limits (mean ± range) by PEEK tubing color, upstream pressure reservoir height, and laboratory**.

| Laboratory | PEEK tubing color | Height (cm) | n |  | Observed color range [min, max] | | | |  | Expanded color range [mean ± range] | | | |
| --- | --- | --- | --- | --- | --- | --- | --- | --- | --- | --- | --- | --- | --- |
|  |  |  |  |  | **Range limits** | **n** | **Inclusion (%)** | **Outliers (%)** |  | **Range limits** | **n** | **Inclusion (%)** | **Outliers (%)** |
| DRF | **Yellow** | **10** | 69 |  | [0.0265, 0.0316] | 30 | 43.5 | 56.5 |  | [0.0232, 0.0335] | 58 | 84.1 | 15.9 |
|  |  | **15** | 15 |  |  | 15 | 100.0 | 0.0 |  |  | 15 | 100.0 | 0.0 |
|  |  | **25** | 70 |  |  | 60 | 85.7 | 14.3 |  |  | 68 | 97.1 | 2.9 |
|  |  | **45** | 70 |  |  | 63 | 90.0 | 10.0 |  |  | 70 | 100.0 | 0.0 |
|  | **Blue** | **10** | 60 |  | [0.0894, 0.1077] | 45 | 75.0 | 25.0 |  | [0.0803, 0.1170] | 60 | 100.0 | 0.0 |
|  |  | **15** | 15 |  |  | 10 | 66.7 | 33.3 |  |  | 14 | 93.3 | 6.7 |
|  |  | **25** | 108 |  |  | 103 | 95.4 | 4.6 |  |  | 106 | 98.1 | 1.9 |
|  |  | **45** | 65 |  |  | 62 | 95.4 | 4.6 |  |  | 65 | 100.0 | 0.0 |
|  | **Orange** | **10** | 65 |  | [1.1731, 1.3476] | 52 | 80.0 | 20.0 |  | [1.0807, 1.4297] | 65 | 100.0 | 0.0 |
|  |  | **15** | 22 |  |  | 21 | 95.5 | 4.5 |  |  | 22 | 100.0 | 0.0 |
|  |  | **25** | 64 |  |  | 64 | 100.0 | 0.0 |  |  | 64 | 100.0 | 0.0 |
|  |  | **45** | 65 |  |  | 65 | 100.0 | 0.0 |  |  | 65 | 100.0 | 0.0 |
| UQAM | **Yellow** | **10** | 45 |  | [0.0240, 0.0307] | 29 | 64.4 | 35.6 |  | [0.0200, 0.0333] | 44 | 97.8 | 2.2 |
|  |  | **25** | 45 |  |  | 41 | 91.1 | 8.9 |  |  | 45 | 100.0 | 0.0 |
|  |  | **45** | 45 |  |  | 45 | 100.0 | 0.0 |  |  | 45 | 100.0 | 0.0 |
|  | **Blue** | **10** | 44 |  | [0.0944, 0.1213] | 39 | 88.6 | 11.4 |  | [0.0769, 0.1307] | 44 | 100.0 | 0.0 |
|  |  | **25** | 45 |  |  | 44 | 97.8 | 2.2 |  |  | 45 | 100.0 | 0.0 |
|  |  | **45** | 43 |  |  | 43 | 100.0 | 0.0 |  |  | 43 | 100.0 | 0.0 |
|  | **Orange** | **10** | 40 |  | [1.3104, 1.5235] | 25 | 62.5 | 37.5 |  | [1.1939, 1.6201] | 40 | 100.0 | 0.0 |
|  |  | **25** | 45 |  |  | 43 | 95.6 | 4.4 |  |  | 45 | 100.0 | 0.0 |
|  |  | **45** | 45 |  |  | 43 | 95.6 | 4.4 |  |  | 45 | 100.0 | 0.0 |
