# Supplementary material for: Low‐Cost Custom‐Built Flow Meters for Plant Hydraulic Conductance: Validation of Accuracy, Precision, and Reproducibility
Source: Plant Direct. 2026 Feb 23;10(2):e70154. doi: 10.1002/pld3.70154 (PMC12928992; doi:10.1002/pld3.70154)
Supplement: Supplementary file 7 — Table S5: Individual PEEK tubing accuracy assessment: Relative deviations by PEEK tubing ID and relative bias by color. [file PLD3-10-e70154-s006.docx]

**Table S5. Accuracy assessment by PEEK tubing ID. For each PEEK tubing ID, mean relative deviation from its reference value was calculated as (measured – reference) / reference × 100%. Weighted mean relative bias was then computed by tubing color, weighted by the number of measurements per ID.**

| Laboratory | PEEK tubing ID | Mean relative deviation by PEEK tubing ID (%) | Weighted mean relative bias by color (%) |
| --- | --- | --- | --- |
| DRF | j1 | -3.31 | -1.89 |
|  | j4 | -2.35 |  |
|  | j5 | 3.17 |  |
|  | j6 | -3.67 |  |
|  | b1 | -2.03 | -0.39 |
|  | b2 | 1.42 |  |
|  | b3 | -3.85 |  |
|  | b4 | 2.03 |  |
|  | o1 | 1.35 | 0.35 |
|  | o2 | 0.13 |  |
|  | o3 | 2.16 |  |
|  | o4 | -1.68 |  |
| UQAM | J1 | 7.93 | 0.16 |
|  | J2 | 1.64 |  |
|  | J3 | -1.37 |  |
|  | J4 | -7.79 |  |
|  | B1 | -8.27 | 1.24 |
|  | B2 | 5.36 |  |
|  | B3 | 2.17 |  |
|  | B4 | 3.68 |  |
|  | O1 | -1.39 | -2.06 |
|  | O2 | -6.66 |  |
|  | O3 | 1.96 |  |
|  | O4 | -0.27 |  |
