# Supplementary material for: Low‐Cost Custom‐Built Flow Meters for Plant Hydraulic Conductance: Validation of Accuracy, Precision, and Reproducibility
Source: Plant Direct. 2026 Feb 23;10(2):e70154. doi: 10.1002/pld3.70154 (PMC12928992; doi:10.1002/pld3.70154)
Supplement: Supplementary file 1 — Data S1: Peer review. [file PLD3-10-e70154-s001.pdf]

Low-cost custom-built flow meters for plant hydraulic conductance: validation of accuracy, precision and reproducibility

Catherine Périé, Jessica Samson-Tshimbalanga , Morgane Urli

Decision Letter Round 1:

November 15, 2025

Mrs. Catherine Périé

Ministere des Ressources naturelles et Forets Secteur des forets

Québec, N/A

Canada

RE: Low-cost custom-built flow meters for plant hydraulic conductance: validation of accuracy, precision and reproducibility

Dear Dr. Périé:

Thank you for submitting to Plant Direct. All required reviews have been returned and we have now finished our evaluation of your manuscript. In light of the reviewers' and editor's comments, further revisions are needed before the paper can be accepted for publication in Plant Direct.

Please view the editors' and reviewers' comments below and use their suggestions as a guide while you work on your revision.

When uploading the revised version of this article, please be sure to include the following:

-A word document that contains your response to the reviewers. You should respond to each reviewer comment and note the changes made to the manuscript. If you do not agree with a reviewer's comment and choose not to make a suggested revision, please explain why. Please try to provide as complete an answer as possible to each reviewer's criticisms.

-A tracked changes document with each change highlighted

- A clean version of the latest version of the manuscript

To upload your revision, please click the link below.

https://plantdirect.msubmit.net/cgi-bin/main.plex?el=A6Lr7py6A6pXj6I3A9ftdqgLmWWNyNAqzHOeKXWQwZ

In order to provide as timely a service as possible, we ask that your revision is resubmitted within three months after receipt of this request. If an extension is needed, please send a request, along with a brief explanation, to the editorial office at plantdirect@wiley.com .

Please note that, in addition to publishing reviewer comments, the author's responses to review comments will also be published alongside the final version of the paper. If you would not like the author's responses to be published, please contact the editorial office at plantdirect@wiley.com .

Thank you very much for giving us an opportunity to review your work. I look forward to receiving the next version.

Sincerely,

Gustavo MacIntosh

Editor, Plant Direct

-- Editor comments:

The reviewers were mostly positive about the manuscript. I agree that adding an example of use of the device with plants will be beneficial. I look forward to receiving a revised version.

-- Reviewer comments:

Reviewer #1:

The authors proposed a low-cost custom-built flow meters for plant hydraulic conductance. The developed method was tested with nearly 1,200 measurements across multiple laboratories, pressure configurations, and tubing resistances, which provides strong statistical support and improves confidence in reproducibility claims. The use of standardized PEEK tubing with known hydraulic properties and reference values derived from displacement measurements is methodologically sound and aligns with prior work (e.g., Melcher et al., 2012; Sack et al., 2011). Moreover, the paper is mostly well-written and clear. Addressing the points below will strengthen the paper and broaden its impact.

1. Although the current validation using PEEK tubing is rigorous, the absence of measurements using real plant stems limits the applicability of conclusions to plant hydraulics research. Even a small demonstration dataset or a discussion of expected artefacts would strengthen the manuscript.

2. The study concludes that accuracy improves above ~25 cm of pressure head based on the PEEK tubing experiment; however, high pressure may alter stem physiology. As the authors acknowledge, elevated pressure could promote embolism displacement. The manuscript would benefit from a more explicit discussion of whether the recommended operating pressure range is biologically safe.

3. Do you have photos of the experimental setups? It would be nice to include it in the supplement for clarity.

Reviewer #2:

This paper is very interesting, the research has both scientific and practical values, for now the paper can be accepted.as this form.

I have only two questions, one is: are there any photos of the experimental devices? two is line 62-63 "Validated flow meters proved 62 portable, affordable (≈ <2500 CAD), and reliable" . How is the cost price calculated?

Decision Letter Round 2:

February 3, 2026

Mrs. Catherine Périé

Ministere des Ressources naturelles et Forets Secteur des forets

Québec, N/A

Canada

MSID: 2025-01855-TWR1

MS TITLE: Low-cost custom-built flow meters for plant hydraulic conductance: validation of accuracy, precision and reproducibility

Dear Dr. Catherine Périé:

I am pleased to inform you that your manuscript "Low-cost custom-built flow meters for plant hydraulic conductance: validation of accuracy, precision and reproducibility" has been accepted for publication in Plant Direct.

Your article will appear online in the next available issue of Plant Direct. To ensure your article gets published as quickly as possible, please pay attention to the steps detailed

below. We have found that most of the delays happen at this stage, especially at the payment stage, so please respond as quickly as possible when prompted.

License Agreement: Once your article has been accepted it will move to Production and undergo admin and file checking - you may receive an email with any queries we have at this stage. When all required items are received by the publisher and queries resolved, the corresponding author will receive an email from Wiley's Author Services system which will ask them to log on at https://authorservices.wiley.com/bauthor and will present them with the appropriate license for completion. Your article cannot be published until both the signed license agreement and payment of the article fee have been received.

Payment of the Open Access Article Publication Fee: All articles published in Plant Direct are fully open access: immediately and freely available to read, download and share. Plant Direct charges a publication fee to cover the publication costs. The corresponding author for this manuscript should have already received a quote from the payments team (cs-openaccess@wiley.com) with the estimated article publication fee; please email cs-openaccess@wiley.com if this has not been received. The corresponding author should log on to the Wiley Author Services site, where the publication fee can be paid by credit card or an invoice. Pro Forma can also be requested. Payment of the publication charge must be received before the article will be published online.

Proofs: You will have the opportunity to look over your paper once more when you receive the author proofs for your article. The proofs will be with you in approximately two weeks. Please note that, in addition to publishing reviewer comments, the author's responses to review comments will also be published alongside the final version of the paper. If you would not like the author's responses to be published, please contact the editorial office at plantdirect@wiley.com .

Promotion of your article: You can help your research get the attention it deserves! Check out Wiley's free Promotion Guide for best-practice recommendations for promoting your work at www.wileyauthors.com/eeo/guide. And learn more about Wiley Editing Services which offers professional video, design, and writing services to create shareable video abstracts, infographics, conference posters, lay summaries, and research news stories for your research at www.wileyauthors.com/eeo/promotion.

Thank you again for your contribution to Plant Direct. If you have any questions, feel free to contact the editorial office at plantdirect@wiley.com .

Sincerely,

Gustavo MacIntosh

Gustavo MacIntosh

Editor, Plant Direct

Response Letter Round 1:

We thank the Editor for the positive evaluation of our manuscript. In response to the comments, we have clarified and strengthened the manuscript by explicitly documenting

the extensive prior use of the same flow meters on plant material, and by expanding the Discussion to address biological considerations related to the applied pressure gradients. These clarifications rely on peer-reviewed evidence and a recently published operational protocol, without adding new experimental data to the present study.

Reviewer #1 – Comments 1 and 2

Comments

1. Although the current validation using PEEK tubing is rigorous, the absence of measurements using real plant stems limits the applicability of conclusions to plant hydraulics research.

2. The study concludes that accuracy improves above ~25 cm of pressure head; however, high pressure may alter stem physiology.

Response

We agree that applicability to biological material and the biological relevance of the operating pressure range are important considerations.

First, we clarified that the flow meters presented in this manuscript correspond to the same custom-built devices developed and routinely used at the Direction de la recherche forestière (DRF), and that these previous version of these devices have

already been applied to excised conifer stems for stem hydraulic conductivity measurements (Urli et al 2023a,b). The operational aspects of the electronic box and the R Shiny application are documented in a dedicated protocol published on Prometheus Protocols (Urli et al., 2025), which is cited in the Methods section.

Second, regarding the applied pressure range, we expanded the Discussion to clarify that the recommended operating conditions are consistent with standard practice in plant hydraulics. The meta-analysis of Urli et al. (2023b) reported extensive conifer stem measurements performed under comparable pressure gradients, and showed no systematic bias in vulnerability curve parameters that would indicate artificial embolism removal.

These points are now clarified in Section 2.1 (Xylem Hydraulic Conductance Measurements Using Custom-built Pressure Drop Flow Meter) and in Section 3.5.1, (Precision analysis).

Reviewer #1 - Comment 3

Do you have photos of the experimental setups?

Response

Yes. Photographs of the experimental setup used in this study have been added to the Supplementary Material, as Fig. S1, to improve clarity.

Reviewer #2 - Comment 1

Are there any photos of the experimental devices?

Response

Yes. Photographs of the experimental setup used in this study have been added to the Supplementary Material, as Fig. S1, to improve clarity.

Reviewer #2 - Comment 2

How is the cost price calculated?

Response

We have clarified in the Methods section how the estimated cost (~2500 CAD) was calculated. The estimate includes the main components required to assemble a functional flow meter (pressure sensors, tubing, and electronic data acquisition components), but excludes labor costs and standard laboratory equipment that are typically already available in most facilities.

Additional editorial revisions

Moreover, several editorial revisions have been made in the revised manuscript. An exhaustive list is provided below:

- L120: Reference updated
- L130-L133: Response to Reviewer #1, Comment 1
- L135: Fig. S1 added in response to Reviewer #1, Comment 3 and Reviewer #2, comment 1
- L147-L148: Citation updated following publication
- L150-L163: Information added in response to Reviewer #2, Comment 2
- L484-L503: Information added in response to Reviewer #1, Comments 1 and 2
- L577: Journal name updated to Plant Direct (initial submission was to JXB)
- L578-L581: Figure S1 legend added
- L596 Two students from Urli lab has been added
- L612-L613: Funding for these two students was added
- L609-L610: Reference updated
- L644-L651: Two references updated
